# The Expandables: Cracking the Staphylococcal Cell Wall for Expansion Microscopy

**DOI:** 10.3389/fcimb.2021.644750

**Published:** 2021-03-16

**Authors:** Tobias C. Kunz, Marcel Rühling, Adriana Moldovan, Kerstin Paprotka, Vera Kozjak-Pavlovic, Thomas Rudel, Martin Fraunholz

**Affiliations:** Department of Microbiology, Julius-Maximilians-Universität Würzburg, Würzburg, Germany

**Keywords:** high-resolution imaging, endosomes, autophagosomes, host-pathogen interaction, expansion microscopy, *Staphylococcus aureus*

## Abstract

Expansion Microscopy (ExM) is a novel tool improving the resolution of fluorescence microscopy by linking the sample into a hydrogel that gets physically expanded in water. Previously, we have used ExM to visualize the intracellular Gram-negative pathogens *Chlamydia trachomatis*, *Simkania negevensis*, and *Neisseria gonorrhoeae*. Gram-positive bacteria have a rigid and thick cell wall that impedes classic expansion strategies. Here we developed an approach, which included a series of enzymatic treatments resulting in isotropic 4× expansion of the Gram-positive pathogen *Staphylococcus aureus.* We further demonstrate the suitability of the technique for imaging of planktonic bacteria as well as endocytosed, intracellular bacteria at a spatial resolution of approximately 60 nm with conventional confocal laser scanning microscopy.

## Introduction

Within the last decade, super-resolution microscopy emerged as a powerful tool for fluorescence-based imaging far beyond the diffraction-limit of conventional light microscopy (Betzig et al., 2006; Hell, 2007; Heilemann et al., 2008). However, due to the high cost of specialized equipment and requirement of special knowledge and experience of the operators, super-resolution methods are mostly restricted to specialized laboratories. Today, most investigations on intracellular pathogens are therefore performed on diffraction-limited conventional microscopy. Expansion microscopy (ExM) is an alternative approach bypassing the diffraction-limit and enabling super-resolution microscopy on standard fluorescence microscopes by physically linking a protein of interest in an expandable hydrogel. Introduced in 2015 (Chen et al., 2015), various ExM protocols were developed leading to sample expansion factors of 4×, 10× (Truckenbrodt et al., 2019) and even 20× (Chang et al., 2017) allowing for high resolution imaging of proteins, nucleotides (Chen et al., 2016) and modified lipids (Gotz et al., 2020a) in cell culture or tissues (Wassie et al., 2019). In addition, expanded samples can be imaged by conventional confocal microscopes or in combination with super-resolution microscopy techniques thereby further improving spatial resolution (Kunz et al., 2020).

ExM has been used to investigate mixed commensal bacterial communities (Lim et al., 2019) and different fungal species (Gotz et al., 2020b). The digestion of fungi thereby required a complex enzymatic mixture, consisting of a lysing enzyme of *Trichoderma harzianum*, driselase and chitinase (Gotz et al., 2020b). Recently, we applied ExM on several pathogens within host cells, such as *Chlamydia trachomatis* (Kunz et al., 2019), *Simkania negevensis*, and *Neisseria gonorrhoeae* (Gotz et al., 2020a). Due to differences in cell wall structure, organism specific digestions strategies are required. Expansion of *C. trachomatis* and *S. negevensis* has been performed with standard expansion protocols using only proteinase K for digestion, while expansion of *N. gonorrhoeae* additionally needed digestion with lysozyme (Kunz et al., 2019; Gotz et al., 2020a). ExM of Gram-positive bacteria was shown to be challenging due to their robust cell envelope. Even though auxiliary digestion steps were introduced into the ExM protocol only 3× expansion was obtained (Lim et al., 2019).

The cell wall of *Staphylococcus aureus*, a Gram-positive human pathogen, possesses a particularly high cross-link ratio between peptidoglycan (PG) chains, which contributes to the structure and the high stability of the bacterial cell wall. Predominantly, these cross-links consist of pentaglycine motifs (Sobral and Tomasz, 2019). Lysostaphin (EC 3.4.24.75) is a staphylolytic endopeptidase and is, for instance, secreted by *Staphylococcus simulans.* Lysostaphin possesses bactericidal properties against several Staphylococcaceae, whereby it specifically cleaves pentaglycine bridges in staphylococcal cell walls (Bastos et al., 2010). Here we show for the first time 4× ExM of planktonic as well as intracellular *S. aureus* by an optimized sample digestion including the combined action of proteinase K, lysozyme and lysostaphin.

## Materials and Methods

### Cell Lines and Bacteria

HeLa229 cells (ATCC CCL-2.1™) were grown at 37°C in a humidified atmosphere containing 5% (v/v) CO_2_ in 10% (v/v) heat inactivated fetal bovine serum (FBS, Sigma-Aldrich) RPMI1640 + GlutaMAX™ medium (Gibco™) complemented with 1 mM sodium pyruvate (Gibco™).

*S. aureus* JE2 (Fey et al., 2013) expressing GFP, encoded by a pJL74 plasmid (Liese et al., 2013), was grown in brain heart infusion medium (BHI, Sigma Aldrich) supplemented with 10 µg/ml erythromycin. An overnight culture was freshly diluted to OD_600 =_ 0.4 in BHI and regrown to exponential phase (OD_600 =_ 0.6–1.0). Bacteria were washed in Dulbeccos´s Phosphate-Buffered Saline (DPBS, ThermoFisher), diluted in infection medium (RPMI Medium with GlutaMAX [ThermoFisher]/10% FBS [Sigma Aldrich]/1 mM sodium pyruvate [ThermoFisher] and then counted in a Thoma chamber. Infection was performed at a multiplicity of infection (MOI) of 10 and was synchronized by centrifugation at 128 *g* for 8 min. For LC3-II staining, infection was performed at a MOI of 5. After 1 h of infection, extracellular bacteria were removed by addition of 20 µg/ml lysostaphin (AMBI) for 30 min. In the case of LC3-II staining, cells were additionally incubated for 1.5 h with 2 µg/ml lysostaphin.

For expansion of planktonic GFP-expressing *S. aureus*, bacteria were grown to exponential phase as described above and collected at an OD_600 _= 5 equivalent by centrifugation at 6,000*g* for 5 min. After subsequent washing with DPBS, bacteria were fixed with 4% paraformaldehyde (PFA) in PBS (Morphisto) for 3 h at room temperature, followed by a slow, 24 h fixation at 7°C.

*C. trachomatis* serovar L2/434/Bu (ATCC VR-902BTM) and *S. negevensis* were propagated in HeLa229 cells at a MOI of 1. The infection was performed for 48 h in the case of *C. trachomatis* and 72 h for *S. negevensis*. Afterwards, the infected cells were detached and lysed by vortexing with glass beads (3 mm, Roth). In order to remove host cell debris, the lysates were centrifuged for 10 min at 4°C and 2000*g* for *C. trachomatis* and 600 g for *S. negevensis*. The supernatant was collected into fresh tubes and subsequently bacteria were pelleted by centrifugation for 30 min at 4°C and 30,000*g* (*C. trachomatis*) or 20,000 g (*S. negevensis*). Bacterial pellets were washed in 1× SPG buffer (7.5% sucrose, 0.052% KH_2_PO_4_, 0.122% NaHPO_4_ and 0.072% L-glutamate), titrated and stored at −80°C for further experiments. Infected host cells were incubated in a humidified atmosphere with 5% (v/v) CO_2_ at 35°C. The cell lines as well as the bacterial stocks were PCR-tested to be free of *Mycoplasm*a.

*N. gonorrhoeae* were cultivated for 16 h at 37°C and 5% CO_2_ on gonococci (GC) agar (Thermo Scientific) plates supplemented with 1% vitamin mix (Bauer et al., 2017). Before infection, the bacteria were cultivated in broth media (protease-peptone medium (PPM) supplemented with 1% vitamin mix and 0.042% sodium bicarbonate) at 37°C and 120 rpm on an orbital shaker. The liquid culture was grown to an OD_550_ of 0.4 to 0.6. Before infection, the bacterial culture was centrifuged for 5 min at 2778 g and the medium was then changed to 4-(2-Hydroxyethyl)piperazine-1-ethanesulfonic acid (HEPES buffer) for infection at MOI 30 for 2 h.

### Antibodies

In this study we used the following primary antibodies and dilutions: Anti-GFP (Abcam, ab1218, dilution 1:100 and OriGene, SP3005P, dilution 1:100), anti-LC3B (Cell Signaling, 2775, dilution 1:100), anti-tubulin (Abcam, ab18251, dilution 1:100 and Sigma Aldrich, T8328, dilution 1:100), anti-lipoteichoic acid (OriGene, BP984, dilution 1:100), anti-LAMP1 (Santa Cruz, H5G11, dilution 1:100), anti-cHSP60 (Santa Cruz, sc-57840, dilution 1:100) and anti-*Neisseria gonorrhoeae* (US biological, N0600-02, dilution 1:200). The secondary antibodies used were Alexa488 conjugated anti rabbit (Thermo Fisher, A-11008, dilution 1:200), Alexa488 conjugated anti mouse (Thermo Fisher, A-11017, dilution 1:200), CF568-cojugated anti rabbit (Sigma Aldrich, SAB4600310, dilution 1:100) and ATTO647N-conjugated anti rabbit (Rockland, 610-156-121S, dilution 1:200).

### Immunostaining and ExM

HeLa229 cells were seeded on 15 mm glass slides (VWR) in multiwell plates (Corning) one day prior to infection. Staining of *S. negevensis* was achieved by membrane staining according to (Gotz et al., 2020a). For immunostaining, the samples were washed in 1× PBS and fixed with 4% PFA in PBS (Morphisto) for 15 min at RT. In the case of LC3-staining, the cells were instead treated with 0.05% saponin and then fixed in MeOH at −20°C for 20 min following a published procedure (Eng et al., 2010). For staining of tubulin, cells were fixed with CB buffer (10 mM 2-(N-morpholino)ethanesulfonic acid (MES), 150 NaCl, 5 mM ethylene glycol-bis(β-aminoethyl ether)tetraacetic acid (EGTA), 5 mM Glucose, 5 mM MgCl_2_, 0.3% GA, 0.25% TX-100, pH 6.1). For all other staining procedures, cells were fixed by 4% PFA in PBS for 15 min at RT. After fixation, the cells infected with *S. aureus* were washed 3× in 1× PBS, permeabilized with 0.2% Triton-X100, treated with 20 µg/ml lysostaphin in PBS for 15 min and then blocked using 10% human serum in 1× PBS (blocking buffer) for 1 h. Cells infected with *C. trachomatis*, *S. negevensis* and *N. gonorrhoeae* were washed 3× in 1× PBS, permeabilized with 0.2% Triton-X100 and blocked using 2% FBS in 1× PBS for 1 h. The cells were then incubated in primary antibody diluted in blocking buffer for 1 h in a humid chamber. For GFP-staining, the incubation was performed over night at 4°C. Afterwards, the samples were washed 3× in 1× PBS and then incubated with the corresponding secondary antibody diluted in blocking buffer for another 1 h. Subsequently, the cells were washed 3× with 1× PBS.

Staining of planktonic *S. aureus* was performed as follows: bacterial cells were fixed with 4% PFA in PBS for 24 h and permeabilized for 20 min in 0.2% Triton-X100 at room temperature, followed by a 1 h treatment at 37°C using 200 µg/mL lysostaphin in 0.1 M phosphate buffer pH 7 (K_2_HPO_4_/KH_2_PO_4_). Cells were then blocked using 10% human serum for 1 h at room temperature and antibody staining was performed using an anti-GFP polyclonal antibody (OriGene Europe, SP3005P, dilution 1:100) for 1 h at room temperature, followed by the incubation with Alexa Fluor 488 secondary antibody (Thermo Fisher, A-11070, dilution 1:100) for 1 h at room temperature. In between each of the above-described steps, samples were washed 3× with 1× PBS and collected by centrifugation at 20,000 g for 1 min. All incubation steps were carried out on an end-over-end rotator (VWR).

### Gelation and Expansion

The expansion protocol was modified from a published procedure for 4× ExM (Chozinski et al., 2016). After immunostaining, infected host cells were incubated for 10 min and planktonic bacteria for 20 min in 0.2% glutaraldehyde (Sigma, G5882) and washed in 1× PBS. For intracellular bacteria, the glass slide was then turned upside down on a drop of the monomer solution (8.625% sodium acrylate, [Sigma, #408220], 2.5% acrylamide [Sigma, #A9926], 0.15% N,N′-methylene bisacrylamide, 2 M NaCl [Sigma, #S5886] in 1× PBS) containing freshly added 0.2% ammonium persulfate (APS; Sigma, A3678) and tetramethyl ethylene diamine (TEMED, Sigma, T7024) for polymerization. Planktonic bacteria were resuspended in monomer solution, pipetted on parafilm and covered with a glass slide. The gel was allowed to polymerize for 90 min at room temperature. The polymerized gel was then removed from the glass slides with tweezers and transferred to digestion buffer (50 mM Tris pH 8.0, 1 mM EDTA [Sigma, ED2P], 0.5% Triton X-100 [ThermoFisher, 28314] and 0.8 M guanidine HCl [Sigma, #50933]), containing lysozyme (5 mg/ml for intracellular and planktonic *S. aureus*, 1 mg/ml for *N. gonorrhoeae*) and lysostaphin (50 µg/ml for intracellular and 200 µg/ml for planktonic *S. aureus*). After 20 min, 8 U/ml protease K (Sigma, P4850) was added for another 30 min. Afterwards, gels were washed and expanded in excess of ddH_2_O. To ensure full expansion, water was exchanged every 60 min until gel size did not further increase. The expanded gels were then cut in smaller pieces of approximately 1–2 cm and transferred to Lab-Tek™ II chambers (VWR, 734-2055) that were coated overnight with 0.1 mg/ml Poly-D-Lysine (ThermoFisher, A3890401).

A step-by-step protocol has been deposited as supplemental online material, which describes the detailed procedure of expansion microscopy adapted for *S. aureus*.

### Microscopy

During acquisition, a drop of water was placed on top of the gels to prevent shrinking due to dehydration of the gels. Confocal imaging was performed on a Leica TCS SP5 (Leica Biosystems, software LAS AF version 2.7.3.9723) using a 63× oil-immersion objective (HCX PL APO lambda blue 63×1.4 NA OIL UV, Leica Biosystems) and a 488 nm Argon laser, 561 nm DPSS laser and a 633 nm HeNe Laser. We recommend decreasing the scanner frequency to 100 Hz to increase signal intensity of expanded samples. SIM-Imaging was done on a Zeiss ELYRA S.1 SR-SIM structured illumination platform (Zeiss, software 12.0.1.362 2012 SP3 [black]) using a 63× water-immersion objective (C-Apochromat, 63×1.2 NA, Zeiss). Reconstruction of SIM-images was performed using the ZEN image-processing platform with a SIM module. Z-stacks were processed using FIJI 1.51n (Schindelin et al., 2012).

### Turbidity Reduction Assays

*S. aureus*-GFP was cultivated over-night in BHI supplemented with 10 µg/ml erythromycin. On the next day, exponential phase cultures (OD_600_ = 0.6–1) were prepared as described before, or stationary phase bacteria were collected. Bacteria were pelleted by centrifugation at 5,000 g for 5 min, washed 1× in PBS and the resulting pellets were resuspended in the appropriate buffers at an adjusted optical density of OD_600_ = 1.0. Turbidity testing was performed in 96-well microtiter plates (Corning, 3596) with samples of a final volume of 200 µl/well in a Tecan Infinite MPlex microtiter plate reader at either 37°C (lysostaphin and mutanolysin) or 25°C (lysozyme). Optical density at 600 nm was measured every 5 min over a course of 1 h with orbital shaking (3.5 mm amplitude) between measurements. In all cases, untreated samples (i.e. bacterial suspension in the appropriate buffer) were used as control to account for spontaneous bacterial lysis. All values were reported as relative to the optical density of the respective condition at the first measured time point (T0). The following enzymes were used in the given final concentration: lysostaphin (50 µg/ml and 200 µg/ml, AMBI 10208), mutanolysin (160 U/ml, Sigma 10501) and lysozyme (1 mg/ml, Sigma 4458). The following buffers were used: Buffer A: 0.1 M Phosphate Buffer pH 7 (K_2_HPO_4_/KH_2_PO_4_); Buffer B: DPBS pH 4.9 (HCl corrected); Buffer C: Digestion Buffer (as described in section “gelation and expansion”).

## Results

**Figure 1:**
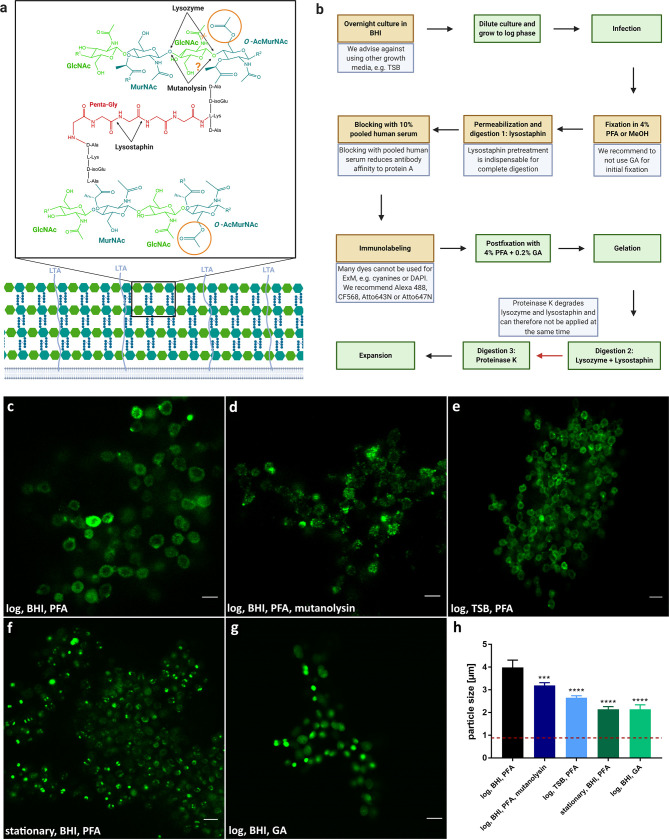
Expansion of *S. aureus* requires several protocol adjustments due to its complex cell wall. **(A)** Structure of staphylococcal cell wall. Peptidoglycan (PG) of *S. aureus* exhibits glycan chains consisting of alternating N-acetyl muramic acid (MurNAc) and N-acetyl glucosamine (GlcNAc) residues, which are linked by β-1,4-glycosidic bonds. Glycan layers are branched by peptides, which possesses a penta-glycine motif specific for *S. aureus*. While lysozyme and mutanolysin specifically cleave β-1,4-linkage between MurNAc and GlcNAc, Lysostaphin digests penta-glycin crosslinks. To prevent degradation by lysozyme and possibly mutanolysin, MurNAc residues in staphylococcal PG are O-acetylated (orange circle). In addition, lipoteichoic acid (LTA) was linked to lysozyme resistance. **(B)** Schematic overview and troubleshooting of sample preparation for expansion of *S. aureus*. Critical steps are indicated in brown or by a red arrow with the respective troubleshooting in blue. Standard steps are shown in green. The protocol for expansion is based on a previous protocol (Chozinski et al., 2016). **(C–G)** Changes in the protocol can affect expansion factor or labeling intensity. *S. aureus* was grown overnight in BHI **(C, D, F, G)** or TSB **(E)**. Then the planktonic bacteria were either diluted and grown to log phase **(C, D, E, G)** or directly used for ExM **(**stationary phase, **F)**. Cultures were fixed using PFA **(C–F)** or GA **(G)**, treated with lysostaphin **(C, E–G)** and mutanolysin **(D)**, permeabilized, immunolabeled for GFP (secondary antibody Alexa488) and expanded. Highest expansion factor (~4×) and best sample quality was obtained in sample **(A)** Scale bars, 5 µm. **(H)** Average particle size of different sample treatments **(C–G)**. Red dotted line indicates size of unexpanded staphylococcal particles (0.88 ± 0.05 µm) for comparison. Significance of the dataset between groups was determined by one-way ANOVA test. Significance between different treatments **(D–G)** to established protocol **(C)** was determined post-hoc by unpaired Student's t-test (***p ≤ 0.0002, ****p ≤ 0.0001).

Bacteria are enveloped by cell walls protecting them from mechanical and physical damage (Vollmer et al., 2008; Vollmer and Seligman, 2010; Sobral and Tomasz, 2019). However, the biological structure of the cell wall and membrane differs strongly between different species. Gram-positive bacteria, for instance, possess a much thicker multi-layered murein sacculus comprised of cross-linked peptidoglycan (PG) layers when compared to PG cell walls of Gram-negative species (e.g. reviewed in (Silhavy et al., 2010)). Independent of Gram-type, PG layers consist of glycan chains of alternating N-acetyl muramic acid (MurNAc) and N-acetyl glucosamine (GlcNAc) residues connected by 1,4-β-glycosidic linkages (**Figure 1A**). PG thus poses a problem for ExM, since the original ExM protocols were developed for eukaryotic cells. Previously, it has been shown that PG has to be digested by lysozyme in order to enable isotropic expansion of bacteria in mixed communities (Lim et al., 2019) or within host cells (Gotz et al., 2020a). In addition, MurNAc residues are modified by peptide chains that are cross-linked connecting individual PG layers (Brott and Clarke, 2019). The PG of *S. aureus* is highly cross-linked by penta-glycine bridges (Vollmer and Seligman, 2010; Sobral and Tomasz, 2019).

We thus explored treatment and digestion protocols in order to enable 4× ExM for the Gram-positive pathogen in the planktonic as well as an intracellular setting. Full expansion was assessed to be complete and isotropic when the bacteria kept their coccoid form and were measured to be ~4× the size of fixed, unexpanded bacteria. We measured an average size of 0.88 +/- 0.05 µm for unexpanded bacteria and 3.40 +/- 0.15, resulting in an expansion factor of 3.85. Expansion of a broad range of extracellular bacteria (Lim et al., 2019) required not only treatment with lysozyme and proteinase K, but also extensive mutanolysin digestion for 20 h at 37°C and pH 4.9. We therefore applied mutanolysin at its pH-optimum of 4.9 to a *S. aureus* culture but did not observe lysis comparable to the lysostaphin treatment during the assayed time (**Supplementary Figure 1**). Extending mutanolysin incubation for fixed samples was unsuitable for expansion of *S. aureus*, since sample integrity was severely compromised during the overnight enzymatic treatment (**Figure 1D**). We therefore explored a protocol that incorporates lysostaphin treatment (**Figure 1C**). Within this protocol, we identified several important steps to enable four-fold expansion (**Figure 1B**): (i) Bacteria have to be grown in BHI medium, since bacteria cultured in the alternative TSB medium only expanded approximately two-fold (**Figure 1E**). (ii) Our protocol enables expansion of *S. aureus* grown in exponential phase, which are also used for host cell infection, whereas *S. aureus* harvested from the stationary phase were only expanded partially (**Figure 1F**). (iii) for sample fixation we recommend using 4% PFA, since fixation with glutaraldehyde (GA) prevented expansion (**Figure 1G**). (iv) Pretreatment with lysostaphin during permeabilization is indispensable for complete digestion. (v) To reduce affinity of antibodies to staphylococcal protein A, samples were blocked with 10% pooled human serum. (vi) Many dyes such as cyanine dyes (e.g. Cy5, Cy3, and Alexa647) cannot be used for ExM, as they are degraded in the polymerization step (Tillberg et al., 2016; Gao et al., 2017). We therefore recommend Alexa488, CF568, Atto643N, or Atto647N (Kunz et al., 2020). (vii) Digestion of the sample after gelation has to be performed in two separate steps, since proteinase K degrades lysozyme and lysostaphin. Therefore, proteinase K is applied after lysozyme and lysostaphin treatment.

**Figure 2:**
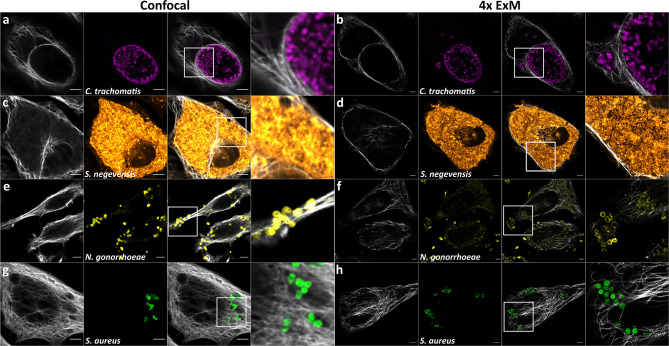
ExM enhances distinguishability of individual intracellular bacterial particles. Confocal fluorescence images of HeLa229 cells infected with **(A, B)** *Chlamydia trachomatis* (anti-cHSP60 and Alexa488, magenta), **(C, D)** *Simkania negevensis* (labeling according to (Gotz et al., 2020a), orange), **(E, F)** *Neisseria gonorrhoeae* (anti-Neisseria and CF568, yellow) or **(G, H)** *Staphylococcus aureus* (anti-GFP and Alexa488, green) before **(A, C, E, G)** and after 4× ExM **(B, D, F, H)**. Infected cells were fixed, permeabilized and labeled for tubulin (secondary antibody **(A–D, G, H)** CF568 or **(E, F)** Alexa488, gray) and the respective pathogen. Scale bars, 10 µm.

Taken together these steps led to full and isotropic expansion of *S. aureus*. In comparison to expansion of the Gram-negative species *C. trachomatis*, *S. negevensis* and *N. gonorrhoeae*, expansion of planktonic *S. aureus* necessitated additional treatment with lysostaphin for digestion of pentaglycine cross-bridges present in the staphylococcal Gram-positive cell wall. (**Figures 1C** and **2**).

**Figure 3:**
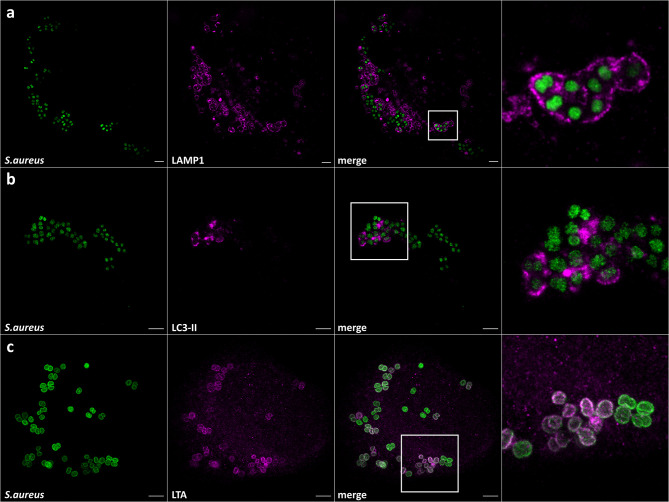
Expansion enables the observation of *S. aureus* in host vesicles and staphylococcal cell wall staining. Confocal images of expanded HeLa229 cells infected with *S. aureus* for 1.5 h (3 h for LC3), fixed, permeabilized and immunolabeled for GFP (secondary antibody **(A)** CF568, **(B, C)** Alexa488, green) and **(A)** LAMP-1 (secondary antibody Alexa488, magenta), **(B)** LC3 (secondary antibody CF568, magenta) and **(C)** lipoteichoic acid (secondary antibody CF568, magenta). Scale bars, 10 µm.

*S. aureus* is a pathogen phagocytosed by professional as well as non-professional phagocytes. The latter include epithelial and endothelial cells, fibroblasts, and keratinocytes (reviewed in Horn et al., 2018). Upon internalization by host cells, *S. aureus* is located in phagosomes or equivalent structures, eventually residing in Lysosomal-associated membrane protein 1 (LAMP-1)-decorated vesicles. In non-professional phagocytes, *S. aureus* is known to escape these vesicles and replicates within the cytosol (reviewed by Horn et al., 2018 and Moldovan and Fraunholz, 2019). Further, it was shown that intracellular *S. aureus* is targeted by xenophagy (Mestre et al., 2010; Mestre and Colombo, 2012; Neumann et al., 2016; Prajsnar et al., 2020) whereby the bacteria are encased by so-called isolation membranes decorated with microtubule-associated proteins 1A/1B light chain 3B (LC3). *S. aureus* seems to be able to exploit autophagosomes as a replicative niche in tissue cells (Schnaith et al., 2007), and even has been shown to survive within LC3-positive vesicles in neutrophils (Prajsnar et al., 2020). We therefore investigated the applicability of ExM to intracellular *S. aureus*, in order to visualize *S. aureus* host interactions with high spatial resolution. For that, we infected HeLa 229 cells with *S. aureus*, whereby *S. aureus* was harvested from exponential growth phase. After 1 h infection pulse, we removed all extracellular bacteria by lysostaphin digestion for 30 min in order to avoid bacterial overgrowth in the medium, which may cause host cell death and immunolabeled for LAMP-1 (**Figure 3A**), LC3 (**Figure 3B**) or lipoteichoic acid (**Figure 3C**). In case of LC3 staining, infection was carried out for additional 1.5 h to ensure the engulfment of a large number of intracellular *S. aureus* by autophagosomes. To prevent extracellular growth of escaped *S. aureus*, the cells were continuously treated with lysostaphin.

**Figure 4:**
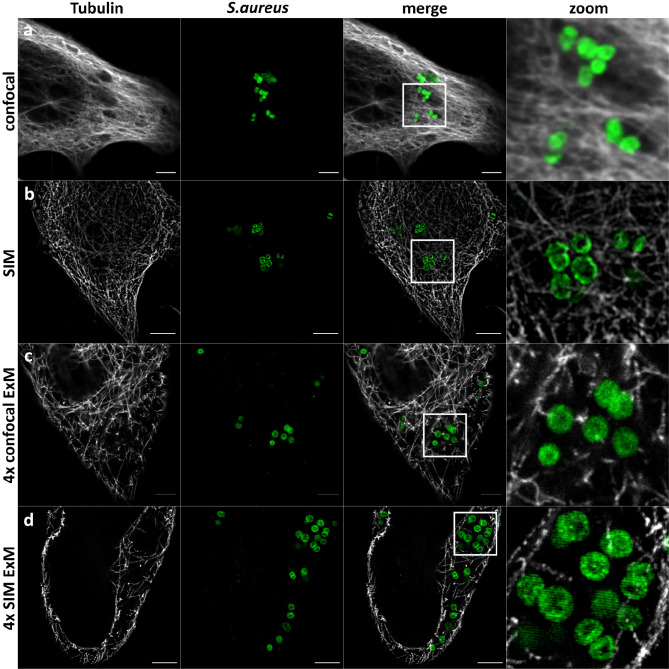
SIM-ExM of *S. aureus* enhances the resolution to ~30 nm. Confocal **(A, C)** and SIM **(B, D)** fluorescence images of HeLa229 cells infected with *S. aureus* (green) before **(A, B)** and after 4× ExM **(C, D)**. Infected cells were fixed, permeabilized and immunolabeled for tubulin (secondary antibody ATTO647N, gray) and GFP (secondary antibody Alexa488, green) and expanded. Scale bars, 5-µm unexpanded and 10-µm expanded samples.

We then embedded the samples in the gel and subjected the samples with the series of enzymatic treatments, which we developed for planktonic *S. aureus* (see above). We expanded the gels and imaged the samples using a confocal laser scanning microscope (CLSM) (**Figure 3**) and structured illumination microscopy (SIM) (**Figure 4**). We thereby fully and isotropically expanded intracellular *S. aureus* and surrounding cell compartments. By CSLM we achieved a resolution of ~60 nm (**Figure 3**), which was further enhanced to ~30 nm by the combination of ExM and SIM (**Figure 4**). We also repeated expansion microscopy with HeLa229 cells infected with the phenotypically agr-negative *S. aureus* strains RN4220 and Cowan I, as well as the strongly cytolytic osteomyelitis strain 6850. All three strains were isotropically expanded by a factor of 4 thereby suggesting that our protocol is generally applicable for different *S. aureus* strains and isolates (**Figure S2**). Hence, by modification of published protocols, we here were able to fully expand planktonic bacteria harvested from the exponential growth phase as well as staphylococci within host cells.

## Discussion

Here, we describe a protocol for ExM of the Gram-positive pathogen *S. aureus*. In comparison to Gram-negative bacteria, *S. aureus* has a more robust and unique cell wall, which features pentaglycine peptides bridging individual peptidoglycan strands. Thus, modifications of the protocol were necessary, since degradation of the bacterial cell wall was found crucial for isotropic expansion (Lim et al., 2019). Our previous expansion of other Gram-negative pathogens such as the members of the Chlamydiaceae, *C. trachomatis* and *S. negevensis*, as well as *N. gonorrhoeae* within host cells did not require modification of the protease regimen (Gotz et al., 2020a). The obligate intracellular *C. trachomatis* does not even possess PG layers although its genome encodes all genes required for PG production (Hesse et al., 2003; McCoy and Maurelli, 2006; Patin et al., 2012). It is currently assumed that *C. trachomatis* produces low amounts of PG only during division (Liechti et al., 2014). Similarly, PG was not identified in the closely related *S. negevensis* (Pilhofer et al., 2013). Hence, we previously demonstrated that the digestion with lysozyme, which is indispensable for other bacteria (Lim et al., 2019) and required for expansion of the pathogen *N. gonorrhoeae* (Gotz et al., 2020a), is not required for both chlamydial species (Kunz et al., 2019; Lim et al., 2019; Gotz et al., 2020a).

In stark contrast, *S. aureus* produces a thick Gram-positive cell wall of peptidoglycan which is not only cross-linked by pentaglycine bridges, but also O-acetylated leading to lysozyme resistance (Brott and Clarke, 2019). O-acetylation of PG also was identified in *N. gonorrhoeae*, which causes infection in genital tract, whereas *S. aureus* normally resides in human nares as a commensal (Schielke et al., 2010; Laux et al., 2019). Lysozyme resistance thereby is important for in-host survival of the bacteria, since lysozyme is produced by macrophages and neutrophils and also is found in several body fluids (Callewaert and Michiels, 2010). Especially in mucus high concentration up to 1 mg/ml were identified (Ragland and Criss, 2017). However, it was previously shown that PG of both species can be degraded by lysozyme, even though with reduced efficiency (Reinicke et al., 1983). The higher amounts of lysozyme required for sufficient digestion of *S. aureus* cell wall in ExM (**Figure 2H**) when compared to that of *N. gonorrhoeae* (**Figure 2F**) thus may result from a higher degree of MurNAc O-acetylation in the *S. aureus* cell wall (~60%) when compared to that of gonococci (34–52%) (Ghuysen and Strominger, 1963; Swim et al., 1983). The O-acetylation ratio of *S. aureus* was shown to also be influenced by chloramphenicol which often is added as selection marker to bacterial culture (Reinicke et al., 1983). However, in our study we used erythromycin selection to maintain episomal GFP expression. In addition, the different thickness of the PG layers between the Gram-positive *S. aureus* (Vollmer and Seligman, 2010) and the Gram-negative *Neisseria* (Pazos and Peters, 2019) may influence muramidase concentration requirements and should be taken into account when adapting the protocol presented here for other organisms.

We also observed a strong dependency of the quality and isotropy of expansion on the growth phase at which staphylococci were harvested. While we were able to fully expand bacteria four-fold when harvested during exponential growth in BHI medium (which also were used for infection of host cells), stationary phase bacteria resisted full expansion (**Figures 1C, F** and **3**). These differences may also relate to the degree of PG O-acetylation, which has been discussed as a maturation process and thus increasing over time by early studies, whereas recent studies did not identify a correlation between O-acetylation ratio between growth phases (Dupont and Clarke, 1991; Bera et al., 2005). Discrepancies between these observations may be explained merely by differences within the investigated bacterial strains and thus differences in the amount of wall teichoic acids (WTA) or D-amino acids in PG. WTA previously were shown to reduce sensitivity of staphylococcal PG against lysozyme (Bera et al., 2007). The incorporation of D-amino acids into the cell wall (Lam et al., 2009; Pidgeon and Pires, 2017) renders PG more resistant to proteases (Horcajo et al., 2012). Hence, stationary phase *S. aureus* overall may resist protease digestion and therefore expansion.

Additionally, we observed that bacteria grown in TSB were more resistant to our digestion procedure and could not be expanded when compared to cultures grown in BHI (**Figures 1C, E** **)**. However, we currently cannot explain this observation other than to imply that the growth medium may influence PG alterations as discussed above. Since both media are routinely used for cultivation of *S. aureu*s, we recommend BHI for bacterial growth in order to support full and isotropic expansion of the pathogen in ExM.

Mutanolysin was found to enhance expansion of several bacteria (Lim et al., 2019). However, the expansion of Gram-positive bacteria remains challenging. In case of *S. aureus*, turbidity assays demonstrated that, similar to lysozyme, mutanolysin did not affect bacterial integrity at relevant incubation times yet compromised staphylococcal structural integrity at longer incubation times (**Figure 1D**). We hypothesize that, comparable to lysozyme, the action of mutanolysin is reduced by the O-acetylation of PG. Thus, mutanolysin was found unsuitable for *S. aureus* ExM. By contrast, lysostaphin led to quick lysis of bacterial cultures **(** **Supplementary Figure 1**) and its application proved crucial for the isotropic 4× expansion of *S. aureus*. Digestion with lysostaphin in combination with lysozyme thereby was required to take place before the proteinase K treatment. We propose that by cleavage of penta-glycine cross-links, the staphylococcal cell wall becomes permeable and renders PG layers accessible for lysozyme. This is supported by reports that show enhancement of lysozyme resistance of *S. aureus* with high PG cross-linkage ratios (Strominger and Ghuysen, 1967; Bera et al., 2007).

Here, we demonstrated that a series of modifications to previously established protocols is required for successful expansion of planktonic *S. aureus* cells or intracellular pathogens. The applicability of our expansion protocol to intracellular *S. aureus* now enables investigation of host-pathogen interaction at resolutions beyond the diffraction limit using standard equipment such as CLSM. We thereby achieved isotropic 4× expansion with a theoretical spatial resolution of 60 nm on a conventional CLSM. This resolution can further be improved by combining ExM with SIM, whereby a lateral resolution of ~30 nm can be achieved. This resolution approaches other super-resolution microscopy methods such as stimulated emission depletion microscopy (STED) (Hell, 2007) or (direct) stochastical optical reconstruction microscopy ((d)STORM) (Heilemann et al., 2008).

*S. aureus* host cell invasion was often described as the crucial aspect for immune evasion, persistency and chronicity of infection (Ou et al., 2017; Horn et al., 2018). We here visualized two organelles with which intracellular *S. aureus* interacts: LAMP1-positive vesicles from which *S. aureus* can escape (reviewed in Moldovan and Fraunholz, 2019) and autophagosomes (**Figure 3**) (Schnaith et al., 2007; Mestre et al., 2010; Mestre and Colombo, 2012; Neumann et al., 2016; Prajsnar et al., 2020). In both cases, ExM enables the clear and highly spatially resolved visualization of *S. aureus* in both LAMP1- and LC3-decorated vesicles. For labeling of bacteria, we used bacteria expressing GFP as well as antibodies directed against lipoteichoic acid. In addition, given the genetic amenability of *S. aureus*, various staphylococcal proteins could be expressed linked to a protein tag for immunolabeling and visualized with ExM at higher resolution. Taken together, ExM provides a powerful method to study host-pathogen interaction and the intracellular virulence mechanisms of *S. aureus*.

## Data Availability Statement

The raw data supporting the conclusions of this article will be made available by the authors, without undue reservation.

## Author Contributions

TCK, MR, and MF conceived the study. TCK, MR, AM, and KP performed the experiments and analyzed the data. VK-P and TR provided strains and reagents. TCK, MR, and MF wrote the manuscript. All authors edited the manuscript. MF supervised the study. All authors contributed to the article and approved the submitted version.

## Funding

This study was supported by the Deutsche Forschungsgemeinschaft (DFG, http://www.dfg.de) within the research training groups RTG 2157 by funds to TR and the RTG 2581 to MF. AM acknowledges funding by the DAAD STIBET Fellowship of the University of Würzburg.

## Conflict of Interest

The authors declare that the research was conducted in the absence of any commercial or financial relationships that could be construed as a potential conflict of interest.

## Supplementary Material

Supplementary File 1 Step-by-step protocol. Detailed procedures for 4× Expansion microscopy of intracellular as well as planktonic *S. aureus*.

Supplementary Figure 1 Lysostaphin, but not lysozyme or mutanolysin lyses *S. aureus* cultures efficiently. *S. aureus* overnight cultures (stationary phase **B, D, F, H**) or log phase grown cultures in BHI medium **(A, C, E, G)** were re-suspended in appropriate buffers and treated with either 1 mg/mL lysozyme **(A, B)**, 160 U/mL mutanolysin **(C, D)**, 50 µg/mL **(E, F)** or 200 µg/mL lysostaphin (g,h). The decrease in culture turbidity was monitored every 5 min, for 1 h, at either 25°C (lysozyme) or 37°C (mutanolysin and lysostaphin). Plots show OD600 reduction relative to the initial inoculation time point (T0). Data points are expressed as mean values, with ± standard deviation (SD); n=3. Treated samples contain the respective enzyme. Untreated samples serve as enzyme-free control. The dashed red line serves as a visual aid marking a 50% reduction in bacterial turbidity as a result of enzymatic activity. A, B, C indicate the respective buffers: Buffer A: 0.1 M Phosphate Buffer pH 7 (K_2_HPO_4_/KH_2_PO_4_); Buffer B: DPBS pH 4.9 (HCl corrected); Buffer C: Digestion Buffer. Statistical analysis is included in **Table S1**.

Supplementary Figure 2 Expansion of other *S. aureus* strains. The laboratory cloning strain *S. aureus* RN4220 **(A)**, as well as *S. aureus s*trains 6850 **(B)** and Cowan I **(C)** were treated as outlined in the manuscript and demonstrated isotropic expansion. This illustrates a general applicability of the used strategy for expanding *S. aureus*. Scale bar 10 μm (expanded sample).

## Abbreviations

APS, ammonium persulfate; BHI, brain heart infusion; cHSP60, chlamydial heat shock protein 60; CLSM, confocal laser scanning microscope; DPBS, Dulbecco’s phosphate-buffered saline; (d)STORM, (direct) stochastical optical reconstruction microscopy; EDTA; EGTA, ethylenediaminetetraacetic acid; ethylene glycol-bis(β-aminoethyl ether)tetraacetic acid; ExM, expansion microscopy; FBS, fetal bovine serum; GA, glutaraldehyde; GFP, green fluorescent protein; GlcNAc, N-acetyl glucosamine; HEPES, 4-(2-hydroxyethyl)piperazine-1-ethanesulfonic acid; LAMP-1, lysosomal-associated membrane protein 1; LC3-II, microtubule-associated proteins 1A/1B light chain 3B; LTA, lipoteichoic acid; MeOH; MES, methanol; 2-(N-morpholino)ethanesulfonic acid; MOI, multiplicity of infection; MurNAc, N-acetyl muramic acid; PCR, polymerase chain reaction; PDL, Poly-D-Lysine; PFA, paraformaldehyde; PG; SD, peptidoglycan; standard deviation; SIM, structured illumination microscopy; STED, stimulated emission depletion microscopy; TEMED, tetramethyl ethylene diamine; TSB, tryptic soy broth; WTA, wall techoic acid.
