# Supplementary material for: The Expandables: Cracking the Staphylococcal Cell Wall for Expansion Microscopy
Source: Front Cell Infect Microbiol. 2021 Mar 16;11:644750. doi: 10.3389/fcimb.2021.644750 (PMC8008081; doi:10.3389/fcimb.2021.644750)
Supplement: Supplementary File 1 — Step-by-step protocol. Detailed procedures for 4× Expansion microscopy of intracellular as well as planktonic S. aureus. [file DataSheet_1.pdf]

Supporting Information

The Expandables: Cracking the staphylococcal cell wall for expansion microscopy

Tobias C. Kunz[1a] & Marcel Rühling[1a], Adriana Moldovan[1], Kerstin Paprotka[1], Vera Kozjak-Pavlovic[1], Thomas Rudel[1], Martin Fraunholz[1b]

[1]Department of Microbiology, Julius-Maximilians-Universität Würzburg, Würzburg, Germany

[a] These authors contributed equally to this work

[b] Correspondence to: martin.fraunholz@uni-wuerzburg.de

Step-by-step protocol for expansion microscopy of *S. aureus*

Reagents

- 4% Paraformaldehyde (PFA, Morphisto, 10303.01000)
- Glutaraldehyde 25% (GA, Sigma Aldrich, G5882)
- Triton X-100 (Thermo Fisher, 28314)
- Fetal bovine serum (FBS, Sigma-Aldrich, F7524)
- Sodium pyruvate (Gibco^{tm}, 11360088)
- TEMED (Sigma, T7024)
- APS (Sigma, A3678)
- PDL (ThermoFisher, A3890401)

Enzymes

- Lysostaphin (AMBI, AMBICINL)
- Lysozyme (Sigma, 62970)
- Proteinase K (Sigma, P4850)

Media

- Infection medium:
  RPMI1640 + GlutaMAX^{TM} (GIBCO, 72400-021)
  10% (v/v) heat inactivated fetal bovine serum (FBS, Sigma-Aldrich, F7524)

  Note: Adjust infection medium to the cell line. This medium was used for Hela229 cells (ATCC CCL-2.1^{TM})

- BHI medium:
  37 g/L of BHI medium (BD, 237300) dissolved in ddH_2O

- TSB agar plates:
  27.5 g/l TSB preparation without dextrose (Sigma, T3938)

15 g/l agar (BD, 257353)
Dissolved in ddH_2O and autoclaved.

Buffers

- 1x DPBS (Thermo Fisher, 14190169)
- 0.1 M phosphate buffer pH 7 (K_2HPO_4/KH_2PO_4, homemade, recipe below)

Phosphate Buffer 1: 1M K_2HPO_4 (174.18 g mol^{-1}) = 87.09 g for 500 ml
Phosphate Buffer 2: 1M KH_2PO_4 (136.09 g mol^{-1}) = 68.045 g for 500 ml
Prepare the 2 buffers separately.
For a 0.1 M phosphate buffer pH 7 mix: 61.5 ml Phosphate Buffer 1 + 38.5 ml Phosphate Buffer 2. Add distilled H_2O ad 1 l. Sterile filter for long-term storage.

- Blocking buffer:
  10% pooled human serum (Sigma, H4522) in DPBS

  Note: S. aureus is natural producer of protein A, a protein with high affinity for IgG from a variety of animal species including humans. Thus blocking with BSA non-fat dry milk etc. is not sufficient to block non-specific binding of fluorescently labelled IgG. Pre-treatment with human serum of commercially available polyglobin eliminates this binding. We advise usage of antisera with low affinity to protein A and to test for non-specific binding in immunofluorescence in advance.

- 4x monomer solution:
  8.625 % Sodium acrylate (Sigma, 408220)
  2.5 % acrylamide (Sigma, A9926)
  0.15 % N, N'-Methylenbisacrylamide (Sigma, A9926)
  2 M NaCl (Sigma, S5886)
  dissolved in 1x DPBS.

- Digestion buffer:
  50 mM Tris pH 8.0 (homemade)
  1 mM EDTA (Sigma, ED2P)
  0.5 % Triton X-100 (Thermo Fisher, 28314)
  0.8 M Guanidine HCl (Sigma, 50933)

Equipment

- Thoma counting chamber (Hartenstein, ZK05)
- 15 mm glass cover slips (0.17 µm thickness, non-fluorescent, VWR, ECN 631-1579)
- Costar 12 well cell culture plates (Corning, 3513)
- Parafilm (Hartenstein, PF10)
- glass chambers (Merck, 734-2055)
- end-over end-rotator (VWR, 444-0500)
- Centrifuge with swing-out plate centrifuge rotor (optional)
- TC dish 150 (Sarstedt, 83.3903)

Procedures for intracellular *S. aureus*

Sample preparation

Day 1:

1. Strike *S. aureus* on an agar plate (TSB) supplemented with antibiotics, if appropriate.

   Note: We recommend using S. aureus strains expressing GFP to enhance signal intensity during imaging. Try to avoid strains with chloramphenicol as a selective marker as it might alter bacterial cell walls (see Discussion in the main manuscript).

Day 2:

2. Seed adherent host cells onto glass cover slips in a 12 well plate. We here seeded 1 x 10^5 HeLa229 per well in infection medium and incubated the cells over night in a humidified atmosphere containing 5% (v/v) CO_2 at 37°C.
3. Prepare an *S. aureus* overnight culture by inoculation of 3 ml BHI medium with a single colony of S. aureus taken from the agar plate (day 1) and grow the culture overnight at 200 rpm and 37°C.

Day 3:

4. Measure the optical density of the bacterial culture at 600 nm (OD_{600}), dilute the culture to an OD_{600} of 0.4 in 10 ml BHI medium and grow the bacteria for approximately 1 hour to reach early exponential growth phase (OD_{600}=0.6-1.0).

   Note: This step originally had been introduced to reset the agr staphylococcal quorum sensing system. agr is activated at high cell densities and causes repression of adhesin production and enhancement of toxin expression. To avoid effects of cytolytic toxins and enhance internalization by non-professional phagocytes it is recommended to dilute overnight cultures of S. aureus accordingly.
   Stationary phase S. aureus resist isotropic expansion with the current protocol (see main manuscript).

5. Harvest bacteria by centrifugation of 1 ml culture at 14.000 rpm for 1 min.
6. Wash 2x with 500 µl DPBS and centrifugation at 14.000 rpm for 1 min.
7. Resuspend the bacterial pellet in 1 ml infection medium and dilute 1:10.
8. Apply 10 µl of the dilution on a Thoma counting chamber.
9. Count bacteria and infect host cells with *S. aureus* at an MOI of 5-10.

   Note: Bacterial number can be established by CFU as well. MOI can be adapted to fit the strain under scrutiny. However, downstream treatments may have to be adapted.

10. Synchronize infection by centrifugation of the plates at 128 g for 8 min in a swing-out plate centrifuge rotor.

    Note: If no synchronization is needed, infection also can be started by sedimentation. The number of intracellular bacteria after the infection pulse and the time of intracellularity then may vary.

11. After 1 h of infection, remove the infection medium and add 300 µl 20 µg/ml lysostaphin (AMBI) in infection medium. Incubate for 30 min in the cell culture incubator. This treatment will remove all extracellular bacteria.

    Note: Lysostaphin specifically cleaves the pentaglycine cross-bridges of S. aureus peptidoglycan. Depending on the specific activity of the enzyme, incubation time and enzyme concentration may have to be adapted.

12. Aspirate off the medium and rinse the cells shortly in 1x DPBS.
13. Aspirate off DPBS and fix the cells by addition of 300 µl 4% PFA in DPBS for 15 min at RT.

    Note: Fixation with MeOH at -20°C for 20 min can improve the labelling with certain antibodies (e.g. anti-LC3B (Cell Signaling, #2775).

14. Wash the samples 3x in 1x DPBS and store overnight at 4°C in 1x DPBS.

Day 4:

Immunostaining

15. Permeabilize and predigest the sample with 0.2% Triton-X100 and 20 µg/ml lysostaphin in DPBS for 15 minutes.
16. Wash 3x in 1x DPBS.
17. Block non-specific antibody binding by addition of 10% pooled human serum in DPBS (blocking buffer) for 1h.

    Note: Staphylococcal protein A non-specifically binds antibodies. Human serum is suitable to block protein A (see above).

18. Incubate samples in primary antibody diluted in blocking buffer for 1h at RT in a humid chamber.

    Note: Proteinase K digestion for expansion degrades GFP. Therefore, when using staphylococcal strains expressing GFP, anti-GFP antibodies need to be applied. Antibodies used in this work are listed in the main manuscript.

19. Wash 3x in 1x DPBS.
20. Incubate the sample in the corresponding secondary antibody diluted in blocking buffer for 1h at RT in a humid chamber in the dark.

    Note: All following steps, including secondary antibody incubation and expansion should be performed with as little light exposure as possible to avoid bleaching.

21. Wash 3x in 1x DPBS.

Sample Expansion

22. Incubate the sample in 0.25% GA at RT for 10 min.

    Note: GA acts as the linker between sample and gel.

23. Wash 3x in 1x DPBS.

24. Freshly prepare 100 µl of the 4x monomer solution per sample by addition of 0.2% ammonium persulfate (APS) and 0.2% tetramethylethylenediamine (TEMED).
25. Immediately pipet 80 µl of the mixture (per cover slip) on a piece of Parafilm and place the cover slips on the drops with the cell sides facing to the polyacrylamide solution.
26. Incubate 90 minutes at RT to polymerize the gel.
27. Remove the gel from the glass slide with a pair of tweezers and transfer it to digestion buffer containing 5 mg/ml lysozyme and 50 µg/ml lysostaphin in a 12 well plate.
28. After 20 minutes, add 8 U/ml proteinase K and incubate at RT for 30 minutes.
29. Wash and transfer the gels in a TC dish 150 in excess of ddH_2O for expansion.
30. Exchange ddH_2O several times until expansion is saturated and leave the gel in ddH_2O overnight.
31. Coat glass chambers with Poly-D-Lysine (PDL) overnight at RT.

Day 5:

32. Cut expanded gels in smaller pieces (around 1 cm^2) that can fit the PDL-coated glass chambers for imaging.

Procedures for planktonic *S. aureus*

Sample preparation

Day 1:

1. Strike out *S. aureus* on a TSB plate supplemented with antibiotics, if appropriate.

   Note: We recommend using S. aureus strains expressing GFP to enhance signal intensity during imaging. Try to avoid strains with chloramphenicol as a selective marker as it might alter bacterial cell walls (see discussion main manuscript).

Day 2:

2. Prepare *S. aureus* overnight culture by inoculation of 3 ml BHI medium and grow overnight at 200 rpm and 37°C.

Day 3:

3. Measure OD_{600}, dilute culture to OD_{600}=0.4 in 10 ml and grow for 1 h to reach exponential phase (OD_{600}=0.6-1.0).

   Note: Stationary phase S. aureus resist isotropic expansion with the current protocol (see main manuscript).

4. Harvest bacteria equivalent to an OD_{600} of 5 by centrifugation at 6.000 g for 5 min.

   Note: OD_{600} equivalent of 5 is equal to OD_{600} in the culture multiplied by culture volume used. Example: 10 ml of a culture with an OD_{600}=0.5 has an OD_{600} equivalent of 5.

5. Wash with DPBS.
6. Fix the bacteria with 4% PFA in DPBS for 3h at RT and subsequently for 24h in a lab refrigerator (6-8°C), protected from light.

Day 4:

Immunostaining

7. Permeabilize the bacteria with 0.2%Triton-X100 in DPBS for 20 min at RT.
8. Wash 3x in 1x DPBS and centrifuge at 20.000 g for 1 min.
9. Predigest the sample with 200 µg/ml lysostaphin for 1h at 37°C in 0.1M phosphate buffer. Alternatively, the 0.1M phosphate buffer can be replaced with DPBS.
10. Wash 3x in 1x DPBS and centrifuge at 20.000 g for 1 min.
11. Block the sample in 10% pooled human serum in DPBS (blocking buffer) for 1h at RT in an end-over-end rotator.

    Note: Staphylococcal protein A unspecifically binds antibodies. Human serum is more suitable to block protein A.

12. Centrifuge at 20.000 g, remove the supernatant and incubate the sample in primary antibody in blocking buffer for 1 h at RT in an end-over-end rotator.

    Note: Proteinase K digestion for expansion degrades GFP. Therefore, when using staphylococcal strains expressing GFP, anti-GFP antibodies need to be applied. Antibodies used in this work are listed in the main manuscript.

13. Wash 3x in 1x DPBS and centrifuge at 20.000 g for 1 min.
14. Incubate the sample in the secondary antibody in blocking buffer for 1 h at RT in an end over end rotator.

    Note: All following steps, including secondary antibody incubation and expansion should be performed with as little light exposure as possible to avoid bleaching.

15. Wash 3x in 1x DPBS and centrifuge at 20.000 g for 1 min.

Sample Expansion

16. Incubate the sample in 0.25% GA in DPBS at RT for 20 min.

    Note: GA acts as the linker between sample and gel.

17. Wash in 1x DPBS and centrifugation at 20.000 g for 1 min.
18. Wash in 4x monomer solution (no APS and TEMED added) and centrifugation at 20.000 g for 1 min.
19. Resuspend pellet in 100 µl 4x monomer solution and add 0.2% ammonium persulfate (APS) and 0.2% tetramethylethylenediamine (TEMED).
20. Immediately pipet 80 µl of the mixture on parafilm and turn a clean 15 mm glass slide upside down onto the drop to form the gel.
21. Incubate 90 minutes at RT to polymerize the gel.
22. Remove the gel from the glass slide with a pair of tweezers and transfer it to digestion buffer containing 5 mg/ml lysozyme and 200 µg/ml lysostaphin in a 12 well plate.

23. After 20 minutes, add 8 U/ml protease K for 30 minutes at RT.
24. Wash and transfer the gels in a TC dish 150 in excess of ddH_2O for expansion.
25. Exchange ddH_2O several times until expansion is saturated and leave the gel in ddH_2O overnight.
26. Coat glass chambers with Poly-D-Lysine (PDL) overnight at RT.

Day 5:

27. Cut expanded gels in smaller pieces (around 1cm^2) that can fit the PDL-coated glass chambers for imaging.
