# Supplementary figures and images for: The Expandables: Cracking the Staphylococcal Cell Wall for Expansion Microscopy
Source: Front Cell Infect Microbiol. 2021 Mar 16;11:644750. doi: 10.3389/fcimb.2021.644750 (PMC8008081; doi:10.3389/fcimb.2021.644750)

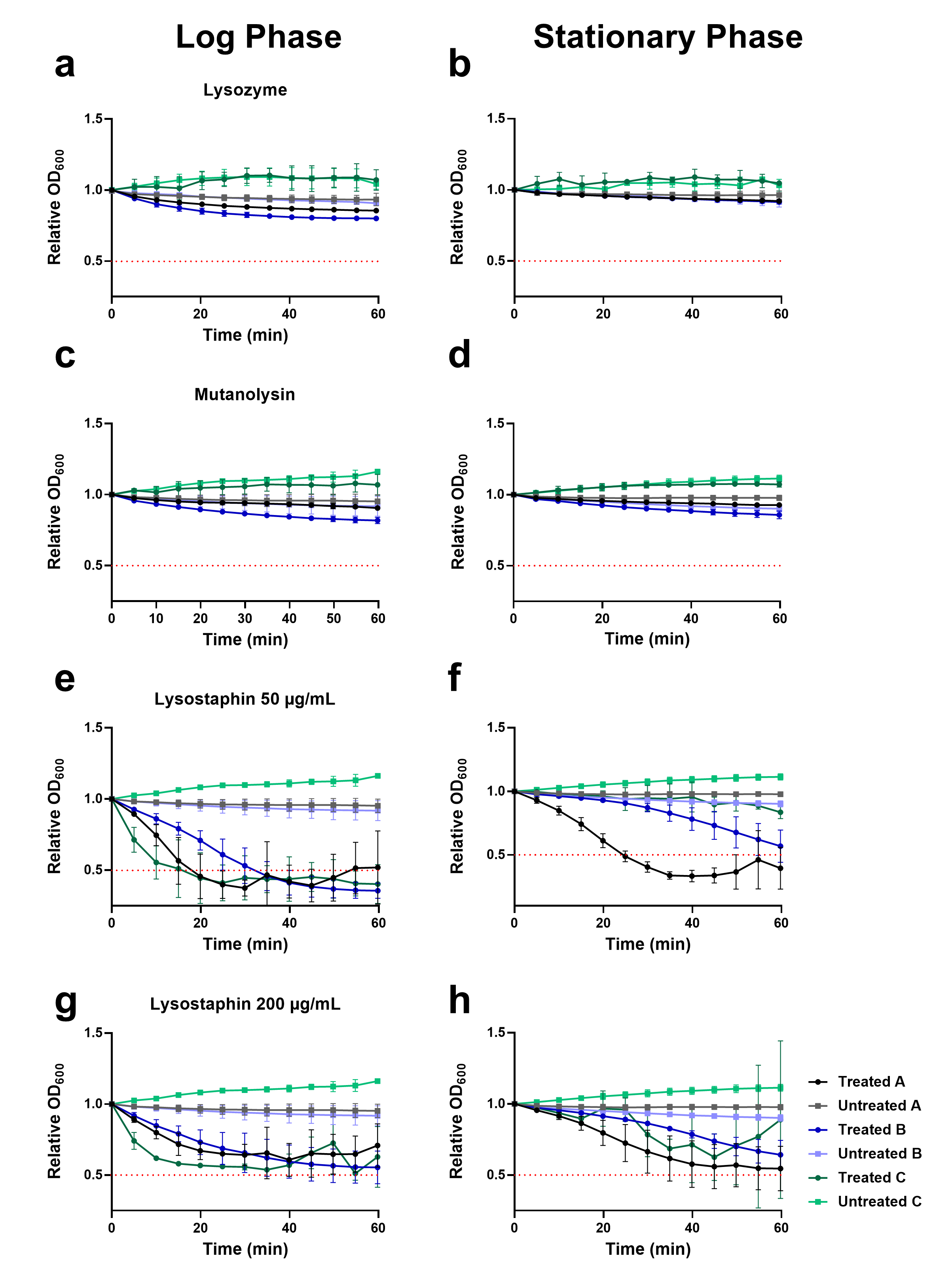

Supplement: Supplementary Figure 1 — Lysostaphin, but not lysozyme or mutanolysin lyses S. aureus cultures efficiently. S. aureus overnight cultures (stationary phase B, D, F, H) or log phase grown cultures in BHI medium (A, C, E, G) were re-suspended in appropriate buffers and treated with either 1 mg/mL lysozyme (A, B), 160 U/mL mutanolysin (C, D), 50 µg/mL (E, F) or 200 µg/mL lysostaphin (g,h). The decrease in culture turbidity was monitored every 5 min, for 1 h, at either 25°C (lysozyme) or 37°C (mutanolysin and lysostaphin). Plots show OD600 reduction relative to the initial inoculation time point (T0). Data points are expressed as mean values, with ± standard deviation (SD); n=3. Treated samples contain the respective enzyme. Untreated samples serve as enzyme-free control. The dashed red line serves as a visual aid marking a 50% reduction in bacterial turbidity as a result of enzymatic activity. A, B, C indicate the respective buffers: Buffer A: 0.1 M Phosphate Buffer pH 7 (K2HPO4/KH2PO4); Buffer B: DPBS pH 4.9 (HCl corrected); Buffer C: Digestion Buffer. Statistical analysis is included in Table S1 . [file Image_1.tif]

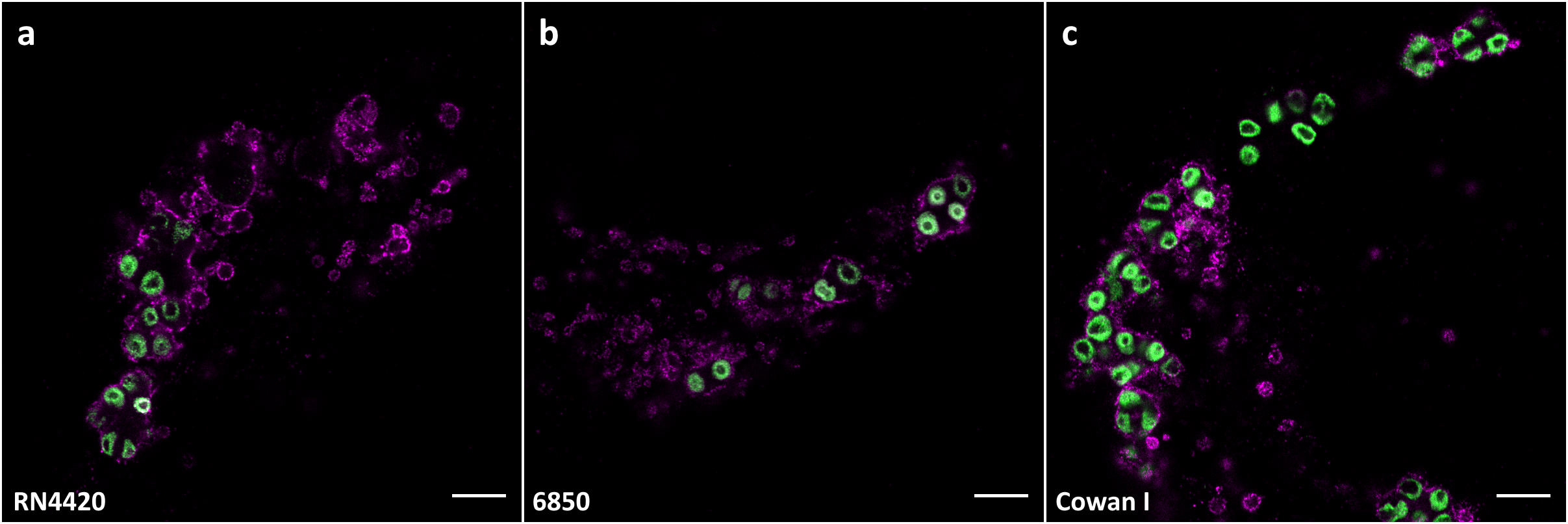

Supplement: Supplementary Figure 2 — Expansion of other S. aureus strains. The laboratory cloning strain S. aureus RN4220 (A), as well as S. aureus strains 6850 (B) and Cowan I (C) were treated as outlined in the manuscript and demonstrated isotropic expansion. This illustrates a general applicability of the used strategy for expanding S. aureus. Scale bar 10 μm (expanded sample). [file Image_2.tif]
