# Supplementary material for: The Expandables: Cracking the Staphylococcal Cell Wall for Expansion Microscopy
Source: Front Cell Infect Microbiol. 2021 Mar 16;11:644750. doi: 10.3389/fcimb.2021.644750 (PMC8008081; doi:10.3389/fcimb.2021.644750)
Supplement: Supplementary file 4 [file Table_1.docx]

|  |  | **Treatment** | | |  |  |  |  |  |  |  |  |  |  |
| --- | --- | --- | --- | --- | --- | --- | --- | --- | --- | --- | --- | --- | --- | --- |
| **Log Phase** | | **Lysozyme** | | |  | **Mutanolysin** | | | **Lysostaphin 50 µg/mL** | | | **Lysostaphin 200 µg/mL** | | |
|  | **Time point (min)** | **A** | **B** | **C** | **A** | **B** |  | **C** | **A** | **B** | **C** | **A** | **B** | **C** |
|  | **0** | **NA** | **NA** | **NA** | **NA** | **NA** |  | **NA** | **NA** | **NA** | **NA** | **NA** | **NA** | **NA** |
|  | **5** | **ns** | **ns** | **ns** | **ns** | **ns** |  | **ns** | ****** | **ns** | **ns** | ***** | **ns** |  |
|  | **10** | **ns** | **ns** | **ns** | **ns** | **ns** |  | **ns** | **ns** | **ns** | **ns** | ***** | **ns** | ******* |
|  | **15** | **ns** | **ns** | **ns** | **ns** | **ns** |  | **ns** | **ns** | ***** | **ns** | **ns** | **ns** | ******** |
|  | **20** | **ns** | **ns** | **ns** | **ns** | **ns** |  | **ns** | **ns** | ***** | **ns** | ***** | **ns** | ******** |
|  | **25** | **ns** | **ns** | **ns** | **ns** | **ns** |  | **ns** | ***** | **ns** | ***** | ***** | **ns** | ******** |
|  | **30** | **ns** | **ns** | **ns** | **ns** | **ns** |  | **ns** | ****** | **ns** | **ns** | ***** | **ns** | ******** |
|  | **35** | **ns** | **ns** | **ns** | **ns** | **ns** |  | **ns** | **ns** | ***** | ***** | **ns** | **ns** | ******** |
|  | **40** | **ns** | **ns** | **ns** | **ns** | **ns** |  | **ns** | ***** | ****** | **ns** | **ns** | **ns** | ***** |
|  | **45** | **ns** | **ns** | **ns** | **ns** | **ns** |  | **ns** | ***** | ****** | ***** | **ns** | **ns** | **ns** |
|  | **50** | **ns** | **ns** | **ns** | **ns** | **ns** |  | **ns** | **ns** | ****** | ***** | **ns** | **ns** | **ns** |
|  | **55** | **ns** | **ns** | **ns** | **ns** | **ns** |  | **ns** | **ns** | ****** | ****** | **ns** | **ns** | ******* |
|  | **60** | **ns** | **ns** | **ns** | **ns** | **ns** |  | **ns** | **ns** | ****** | ***** | **ns** | **ns** | **ns** |
| **Stationary Phase** | |  |  |  |  |  |  |  |  |  |  |  |  |  |
|  | **0** | **NA** | **NA** | **NA** | **NA** | **NA** |  | **NA** | **NA** | **NA** | **NA** | **NA** | **NA** | **NA** |
|  | **5** | **ns** | **ns** | **ns** | **ns** | **ns** |  | **ns** | **ns** | **ns** | **ns** | **ns** | **ns** | **ns** |
|  | **10** | **ns** | **ns** | **ns** | **ns** | **ns** |  | **ns** | **ns** | **ns** | **ns** | **ns** | **ns** | **ns** |
|  | **15** | **ns** | **ns** | **ns** | **ns** | **ns** |  | **ns** | ***** | **ns** | **ns** | **ns** | **ns** | ***** |
|  | **20** | **ns** | **ns** | **ns** | **ns** | **ns** |  | **ns** | ***** | **ns** | **ns** | **ns** | ***** | **ns** |
|  | **25** | **ns** | **ns** | **ns** | **ns** | **ns** |  | **ns** | ****** | **ns** | **ns** | **ns** | ****** | **ns** |
|  | **30** | **ns** | **ns** | **ns** | **ns** | **ns** |  | **ns** | ****** | **ns** | **ns** | **ns** | ****** | **ns** |
|  | **35** | **ns** | **ns** | **ns** | **ns** | **ns** |  | **ns** | ******* | **ns** | **ns** | **ns** | ****** | ***** |
|  | **40** | **ns** | **ns** | **ns** | **ns** | **ns** |  | **ns** | ****** | **ns** | **ns** | **ns** | ***** | **ns** |
|  | **45** | **ns** | **ns** | **ns** | **ns** | **ns** |  | **ns** | ****** | **ns** | **ns** | **ns** | **ns** | **ns** |
|  | **50** | **ns** | **ns** | **ns** | **ns** | **ns** |  | **ns** | **ns** | **ns** | **ns** | **ns** | **ns** | **ns** |
|  | **55** | **ns** | **ns** | **ns** | **ns** | **ns** |  | **ns** | **ns** | **ns** | ****** | **ns** | **ns** | **ns** |
|  | **60** | **ns** | **ns** | **ns** | **ns** | **ns** |  | **ns** | **ns** | **ns** | ***** | **ns** | **ns** | **ns** |

**Table S1** Statistical analysis summary of the results corresponding to Fig S1

Samples corresponding to **one condition** (eg. Lysozyme log phase) were analysed separately by 2way ANOVA with Geisser-Greenhouse correction (no assumption of sphericity) followed by Tukey’s multiple comparisons test. Table summarises the results of comparing, for each tested buffer (A, B or C), the **treated samples** vs **untreated sample**, in the same buffer, **at** **each represented time point** (**P<0.05; **P<0.01; ***P<0.001; ****P<0.0001; ns=not significant*). NA=not applicable. Statistical analysis was performed using GraphPad Prism (version 9.0.0).
